# Cysteine-rich receptor-like secreted protein 1 promotes intercellular infection and enhances nodulation in *Aeschynomene indica*

**DOI:** 10.1093/hr/uhaf185

**Published:** 2025-07-22

**Authors:** Zeming Huang, Guiling Ren, Xijie Guo, Yaxing Su, Yuchen Wang, Shuwen Zhang, Xingjiang Qi, Huijie Lu, Jiazhang Lian, Yan Liang

**Affiliations:** Zhejiang Provincial Key Laboratory of Agricultural Microbiomics, Key Laboratory for Agricultural Microbiome of the Ministry of Agriculture and Rural Affairs, Institute of Biotechnology, Zhejiang University, 866 Yuhangtang Road, Xihu District, Hangzhou 310058, China; Zhejiang Provincial Key Laboratory of Agricultural Microbiomics, Key Laboratory for Agricultural Microbiome of the Ministry of Agriculture and Rural Affairs, Institute of Biotechnology, Zhejiang University, 866 Yuhangtang Road, Xihu District, Hangzhou 310058, China; Zhejiang Provincial Key Laboratory of Agricultural Microbiomics, Key Laboratory for Agricultural Microbiome of the Ministry of Agriculture and Rural Affairs, Institute of Biotechnology, Zhejiang University, 866 Yuhangtang Road, Xihu District, Hangzhou 310058, China; Zhejiang Provincial Key Laboratory of Agricultural Microbiomics, Key Laboratory for Agricultural Microbiome of the Ministry of Agriculture and Rural Affairs, Institute of Biotechnology, Zhejiang University, 866 Yuhangtang Road, Xihu District, Hangzhou 310058, China; Zhejiang Provincial Key Laboratory of Agricultural Microbiomics, Key Laboratory for Agricultural Microbiome of the Ministry of Agriculture and Rural Affairs, Institute of Biotechnology, Zhejiang University, 866 Yuhangtang Road, Xihu District, Hangzhou 310058, China; Institute of Horticulture, Zhejiang Academy of Agricultural Sciences, 298 Desheng Mid Road, Xihu District, Hangzhou 310021, China; Institute of Horticulture, Zhejiang Academy of Agricultural Sciences, 298 Desheng Mid Road, Xihu District, Hangzhou 310021, China; Department of Environmental Engineering, College of Environmental and Resource Sciences, State Key Laboratory of Soil Pollution Control and Safety, Zhejiang University, 866 Yuhangtang Road, Xihu District, Hangzhou 310058, China; Institute of Bioengineering, College of Chemical and Biological Engineering, Zhejiang University, 866 Yuhangtang Road, Xihu District, Hangzhou 310058, China; Zhejiang Provincial Key Laboratory of Agricultural Microbiomics, Key Laboratory for Agricultural Microbiome of the Ministry of Agriculture and Rural Affairs, Institute of Biotechnology, Zhejiang University, 866 Yuhangtang Road, Xihu District, Hangzhou 310058, China

## Abstract

Nitrogen-fixing bacteria establish symbiotic relationships with their host plants via two different entry systems: root hair-mediated (intracellular) entry and intercellular entry. However, the molecular mechanisms underlying the intercellular entry system have received relatively little research attention. In this study, we compared the transcriptomes of the nodules and roots of *Myrica rubra*, which forms an ancient type of symbiosis with *Frankia* via intercellular entry*.* We found that *cysteine-rich receptor-like secreted protein 1* (*CRRSP1*) was highly upregulated in *M. rubra* nodules. We then investigated the function of *MrCRRSP1* in *Aeschynomene indica,* which establishes symbiosis with *Bradyrhizobium* sp. ORS285 through an intercellular entry system. The overexpression of *MrCRRSP1* and *AiCRRSP1* in *A. indica* enhanced the nodule number and plant growth. Exogenous application of glutathione S-transferase (GST)-tagged MrCRRSP1 and AiCRRSP1 in *A. indica* promoted rhizobial attachment at cracks in the lateral root base, as well as rhizobial motility and biofilm formation. These results suggest that CRRSP1 promotes nodulation by enhancing rhizobial attachment to lateral root cracks. In addition to providing new insights into the molecular mechanisms underlying nodule formation through intercellular entry, this research enhances our understanding of actinorhizal plant–*Frankia* symbiosis.

## Introduction

Biological nitrogen fixation is an ecologically important process that plays a crucial role in various aspects of agriculture. Fixation is performed by prokaryotic species that contain nitrogenase, an enzyme capable of converting atmospheric nitrogen into ammonia [1]. The most efficient biological nitrogen fixation process is symbiotic nitrogen fixation, which includes legume–rhizobia and actinorhizal plant–*Frankia* symbioses [2]. These symbiotic relationships give rise to a unique organ known as the nodule, which provides specialized low-oxygen conditions for nitrogenase enzymes [3]. Inside the nodules, bacteria supply the plant with nitrogen in exchange for carbon sources provided by the plant [4].

Most legume-type nodules are initiated through a dialogue between plants and bacteria. Flavonoids secreted by legumes provoke rhizobia to produce lipochitooligosaccharide (LCO) compounds, the so-called Nod factors [5]. Recognition of Nod factors by legume receptors triggers a complex cascade of signal transduction processes, referred to as common symbiotic pathways that lead to a series of morphological changes within the plant [1]. Specifically, the root hair curls and entraps bacteria, prompting the formation of an infection thread that guides the bacteria toward the plant root cortex [6]. Concurrently, cortical cells undergo redifferentiation to form the nodule primordium, which eventually develops into mature nodules. This root hair-mediated invasion system, known as intracellular infection, is stringently regulated by the Nod factor signaling pathway [7]. The majority of leguminous species form symbiotic nodules via the intracellular infection pathway [1]. In contrast, certain legumes (e.g. *Arachis*, *Aeschynomene*, *Sesbania*) and many actinorhizal plants employ an intercellular infection mechanism [8]. In this alternative pathway, bacteria penetrate the roots via cracks in the lateral root base or the middle lamellae between two adjacent root hair cells [9]. Notably, certain legumes exhibit conditional intercellular infection. For instance, two *Lotus* species (*Lotus burttii* and *Lotus japonicus*) can be colonized intercellularly by specific rhizobial strains, including *Sinorhizobium fredii*, *Rhizobium leguminosarum*, and *Agrobacterium pusense* IRBG74 [10, 11].

Actinorhizal plant–*Frankia* symbiosis is found within eight lineages of plant species belonging to three orders: Fagales, Cucurbitales, and Rosales [12]. A key difference between actinorhizal and legume nodules is their distinctive structural arrangement [2]. Legume nodules exhibit stem-like shapes with a vascular system at the periphery, whereas actinorhizal nodules resemble roots with a vascular system at the center. Nonetheless, when considering infected cells that harbor intracellular nitrogen-fixing microsymbionts, all originate from the root cortex [13]. Furthermore, although the signaling molecules involved in actinorhizal plant–*Frankia* symbiosis are yet to be identified, the common symbiosis pathway is conserved among actinorhizal plant species [14, 15]. Consequently, researchers have proposed that actinorhizal-type nodules are ancestral, whereas legume-type nodules evolved from them [13]. Compared to legume–rhizobia symbiosis, our understanding of actinorhizal plant–*Frankia* symbiosis is relatively limited.

Among the actinorhizal plants capable of forming symbiotic relationships with *Frankia*, the Myricaceae family is the oldest [16]. The Chinese bayberry (*Myrica rubra*), a member of the Myricaceae family, is a tropical or subtropical tree native to East Asia, predominantly found in southern China [17]. Chinese bayberries are highly prized for their beautiful taste, appealing color, and significance in traditional Chinese medicine. Consequently, the genome and transcriptome of Chinese bayberries have been thoroughly documented [17], thereby facilitating the investigation of symbiotic events in the ancient actinorhizal plant–*Frankia* system.

In this study, we examined the nodule structure of *M. rubra* and compared the differential transcripts present in nodules and roots. Genes involved in the common symbiotic pathway were significantly upregulated in *M. rubra* nodules. Notably, four cysteine-rich receptor-like secreted proteins (CRRSPs) were highly upregulated in *M. rubra* nodules. The overexpression (OE) of *MrCRRSP1* in *Aeschynomene indica* enhanced the number of nodules, and the application of prokaryotically expressed MrCRRSP1 and AiCRRSP1 promoted bacterial colonization at the cracks of the lateral root base. Our results suggest that CRRSP1 might play a role in intercellular infection.

## Results

### *Myrica rubra* develops lateral root-like nodules

**Figure 1:**
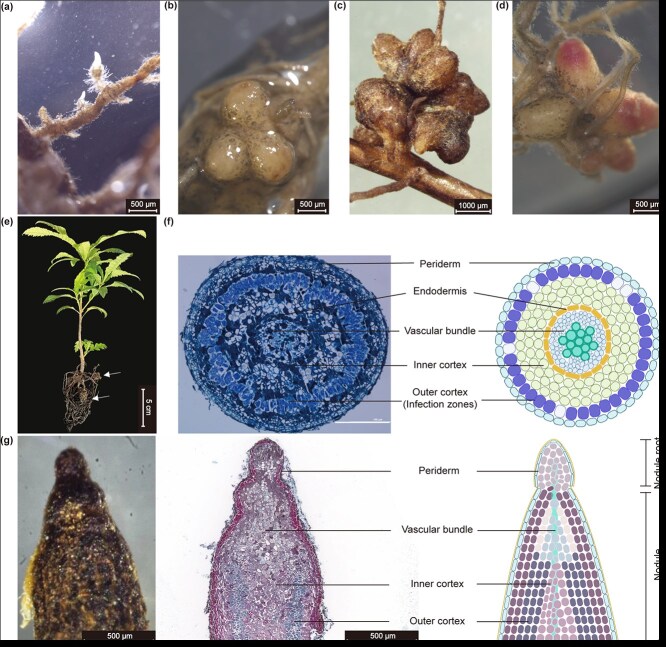
*Myrica rubra* develops lateral root-like nodules. (a) A potential future nodulation root featuring an enhanced density of root hairs. The image was captured at 3 wpi. (b) Nodules at 4–5 wpi. (c) Nodules at 12 wpi. (d) Nodules with pink tips. (e) Six-month-old *M. rubra* seedlings. Arrows indicate nodules. (f) Transverse section anatomy of nodule (left panel) and its schematic diagram (right panel). Five-week-old *M. rubra* nodules were cross-sectioned and stained with toluidine blue. The nodule’s structure, from the outer to the inner layers, comprises the periderm, outer cortex, inner cortex, and the central vascular system. (g) Nodule with a root on the tip (left panel), its longitudinal section (middle panel) and schematic diagram (right panel). Bars: (a, b, d, g) 500 μm; (c) 1000 μm; (e) 5 cm; (f) 100 μm.

To observe the early development of nodules in *M. rubra* roots, 2-month-old seedlings were inoculated with homogenized suspensions derived from mature *M. rubra* nodules. At 3 weeks postinoculation (wpi), certain roots exhibited increased growth of lateral roots and root hairs, suggesting potential future nodulation (Fig. 1a). By 4–5 wpi, nodules with a few lobes were found on the newly developed roots (Fig. 1b); by 12 wpi, multilobed nodules had formed (Fig. 1c). Young nodules displayed a white or light-yellow color, occasionally featuring a pink tip (Fig. 1d), whereas mature nodules eventually turned blackish-brown and gathered in clusters (Fig. 1e). The dimensions of a single mature nodule varied from 1.00 to 1.50 mm in length and 0.75–1.00 mm in width (Fig. 1c–e).

The transverse section of 5-week-old *M. rubra* nodules revealed an elliptical structure with the vascular system at the center of the nodule, mimicking lateral root development (Fig. 1f). The outer cortex, characterized by a spindle-shaped structure, was densely populated with numerous infected cells. In contrast, the inner cortex and vascular tissues showed a marked reduction in the number of infected cells (Fig. 1f). Moreover, the volume of infected cells was larger than that of noninfected cells (Fig. 1f). Occasionally, root structures were observed on the nodules, and a longitudinal section verified the emergence of new roots from the tips of the nodules (Fig. 1g). Overall, the morphological and anatomical observations suggest that *M. rubra* develops lateral root-like nodules.

### Global analysis of gene expression in *M. rubra* nodules

**Figure 2:**
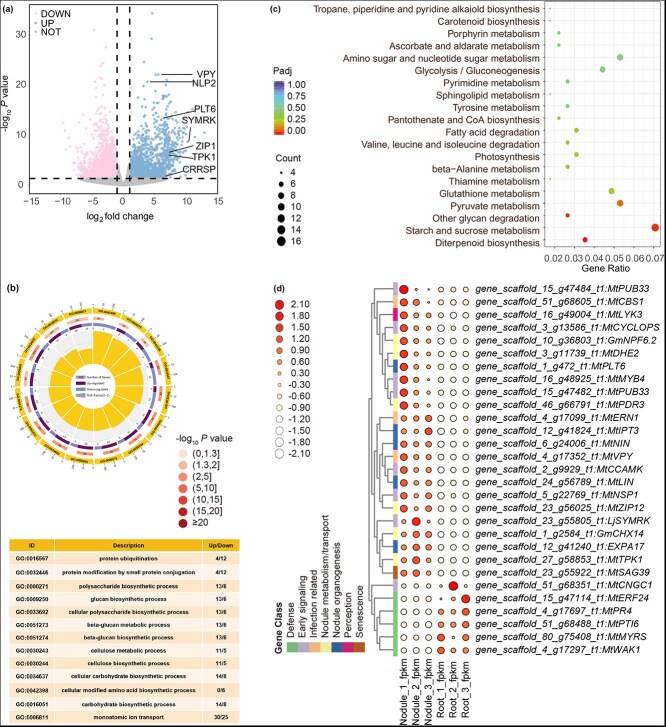
Global analysis of gene expression in *M. rubra* nodules. (a) Volcano plot showing the DEGs between nodules and roots. The significantly upregulated and downregulated DEGs are shown. (b) Functional enrichment analysis of DEGs in *M. rubra* nodules. The top 16 enriched Gene Ontology terms are shown. (c) Kyoto Encyclopedia of Genes and Genomes pathway enrichment analysis showing significantly upregulated metabolic pathways in *M. rubra* nodules. (d) Heatmap plot showing DEGs involved in nitrogen-fixing symbiosis. The symbiotic genes in *M. truncatula*, *Glycine max*, and *L. japonicus* serve as references for identifying orthologs in *M. rubra*.

To understand early gene regulation in *M. rubra* nodules, RNA sequencing analysis was performed using 2-month-old nodules and the roots where nodules were located. We identified 3052 differentially expressed genes (DEGs, |log2FoldChange| >1.0, *P*_adj_ < 0.05) with 1482 upregulated and 1570 downregulated genes in the nodules compared to the roots (Fig. 2a and Table S1). Gene ontology enrichment analysis revealed that the top upregulated DEGs in the nodules were involved in polysaccharide biosynthetic processes, glucan biosynthetic processes, cellulose biosynthetic processes, and monoatomic ion transport, whereas the top downregulated DEGs were related to cellular modified amino acid biosynthetic processes, protein modification by small protein conjugation, and protein ubiquitination processes (Fig. 2b). Kyoto Encyclopedia of Genes and Genomes pathway analysis indicated that polysaccharide biosynthesis and metabolic processes were significantly upregulated in *M. rubra* nodules (Fig. 2c). In addition, the diterpenoid biosynthesis pathway, including genes involved in gibberellic acid biosynthesis, was highly upregulated in *M. rubra* nodules, whereas defense-related pathways were downregulated (Fig. 2d). We then identified the DEGs involved in symbiosis in legumes and actinorhizal plants (Fig. 2d and Table S2). Conserved nodule development genes, such as *NODULE INCEPTION (NIN)* and *NODULATION SIGNALING PATHWAY* (*NSP1* and *NSP2*) were upregulated in *M. rubra* nodules (Fig. 2d). Early symbiosis signaling, including *SYMBIOSIS RECEPTOR KINASE (SYMRK)* and *CALCIUM AND CALMODULIN-DEPENDENT PROTEIN KINASE (CCAMK)* were also identified in *M. rubra* nodules (Fig. 2d). Together, these results suggest that the genes involved in nodule development and common symbiotic signaling pathways are conserved in *M. rubra*.

### Identification of putative Nod factor perception (NFP) in *M. rubra*

**Figure 3:**
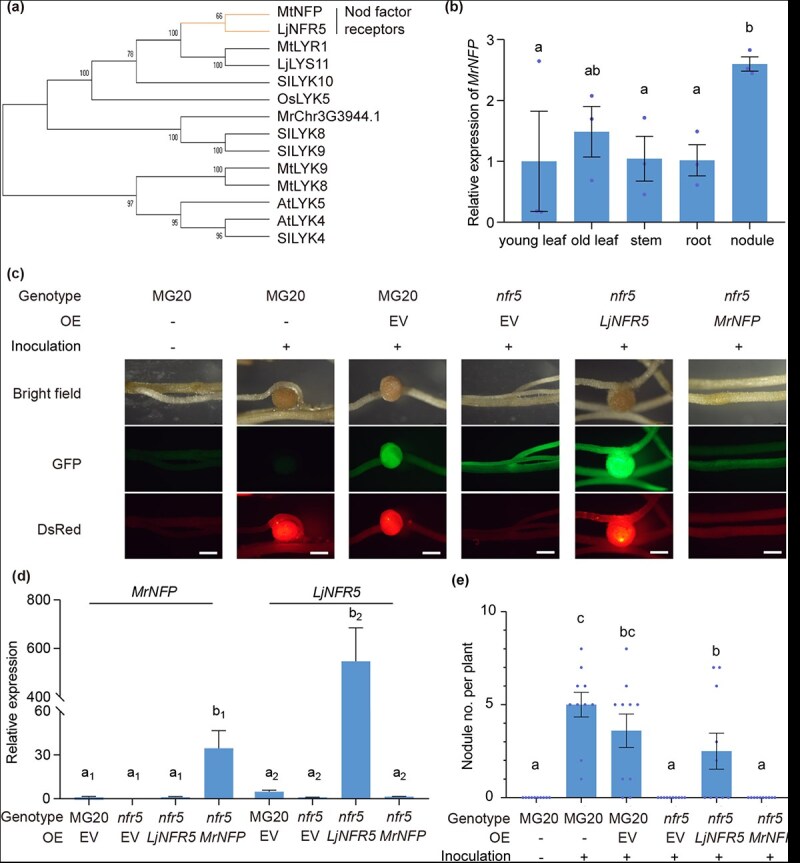
OE of *MrNFP* did not restore the nodulation of *Ljnfr5* mutants. (a) Phylogenetic tree of NFP subclade. *Mt, M. truncatula; Lj, L. japonicus; Sl, S. lycopersicum; Os, O. sativa*. (b) The expression level of *MrNFP* in various tissues of *M. rubra*. (c–e) OE of *MrNFP* and *LjNFR5* in *Ljnfr5* mutants via hairy root transformation. Representative images are shown in (c). Fluorescence signals represent transgenic roots expressing fluorescent proteins (middle panel) and nodules harboring DsRed-labeled *M. loti* (bottom panel). EV, empty vector; MG20, the background of *Ljnfr5* mutant; Bars, 500 μm. The relative expression levels of transgenes were detected by RT-qPCR (d) and nodule number was counted at 4 wpi (e). Data are means ± SE (*n* = 3 in b, d, and 10 in e). Different letters above the bars indicate significant differences between different groups (*P* ≤ 0.05, one-way ANOVA).

The common symbiotic signaling pathway is initiated by LCO receptors in legumes for nodulation, all of which belong to the lysin motif receptor-like kinase (LYK) family. Therefore, we identified *LYK* members in the *M. rubra* genome and detected 13 candidates. LYKs contain conserved structural elements including extracellular lysin motifs, transmembrane domains, and intracellular kinase domains. We then performed phylogenetic analysis using the amino acid sequences of LYKs from *Medicago truncatula*, *L. japonicus*, *Solanum lycopersicum*, *Oryza sativa,* and *M. rubra* (Fig. S1). In *M. rubra*, MrChr3G3944.1 falls into the LjNFR5/MtNFP clade (Fig. 3a) and is renamed MrNFP hereafter. The expression level of *MrNFP* was slightly higher in the nodules compared to that in the leaves and roots (Fig. 3b).

To examine whether MrNFP can initiate the LCO-induced common symbiotic signaling pathway, we overexpressed *MrNFP* in *Ljnfr5* mutants using the *Agrobacterium rhizogene*-mediated hairy root transformation method, with *LjNFR5* as a control (Fig. 3c). Reverse transcription quantitative PCR (RT-qPCR) confirmed the OE of *MrNFP* and *LjNFR5* (Fig. 3d). Our results indicated that *LjNFR5* OE restored nodule formation in *Ljnfr5* mutants, whereas no nodules were observed in roots where *MrNFP* was overexpressed in a *Ljnfr5* background (Fig. 3e). These results suggest that the ectopic expression of *MrNFP* does not complement the function of *LjNFR5* in LCO recognition during nodule development in *L. japonicus*.

NFP duplication may occur within the nitrogen-fixing clade, with the NFP-II-type gene evolving to function as an LCO receptor, whereas the NFP-I-type lacks this capability [18]. In the phylogenetic reconstruction of NFP family, which included legumes and actinorhizal species, we noticed that MrNFP was not present in the subclade alongside *Alnus glutinosa* and *Casuarina glauca,* two actinorhizal species nodulated by *Frankia* spp.; instead, MrNFP was categorized outside the nitrogen-fixing clade (Fig. S2). In addition, MrNFP did not contain the residues L118, N122, S148, or P153 (Fig. S3), which represent the LCO-binding sites of LjNFR5 in *L. japonicus* or MtNFP in *M. truncatula* [19, 20]. These results suggest that LCO recognition might not be required for nodulation in *M. rubra.*

### Characterization of cysteine-rich receptor-like secreted protein 1 in *M. rubra*

**Figure 4:**
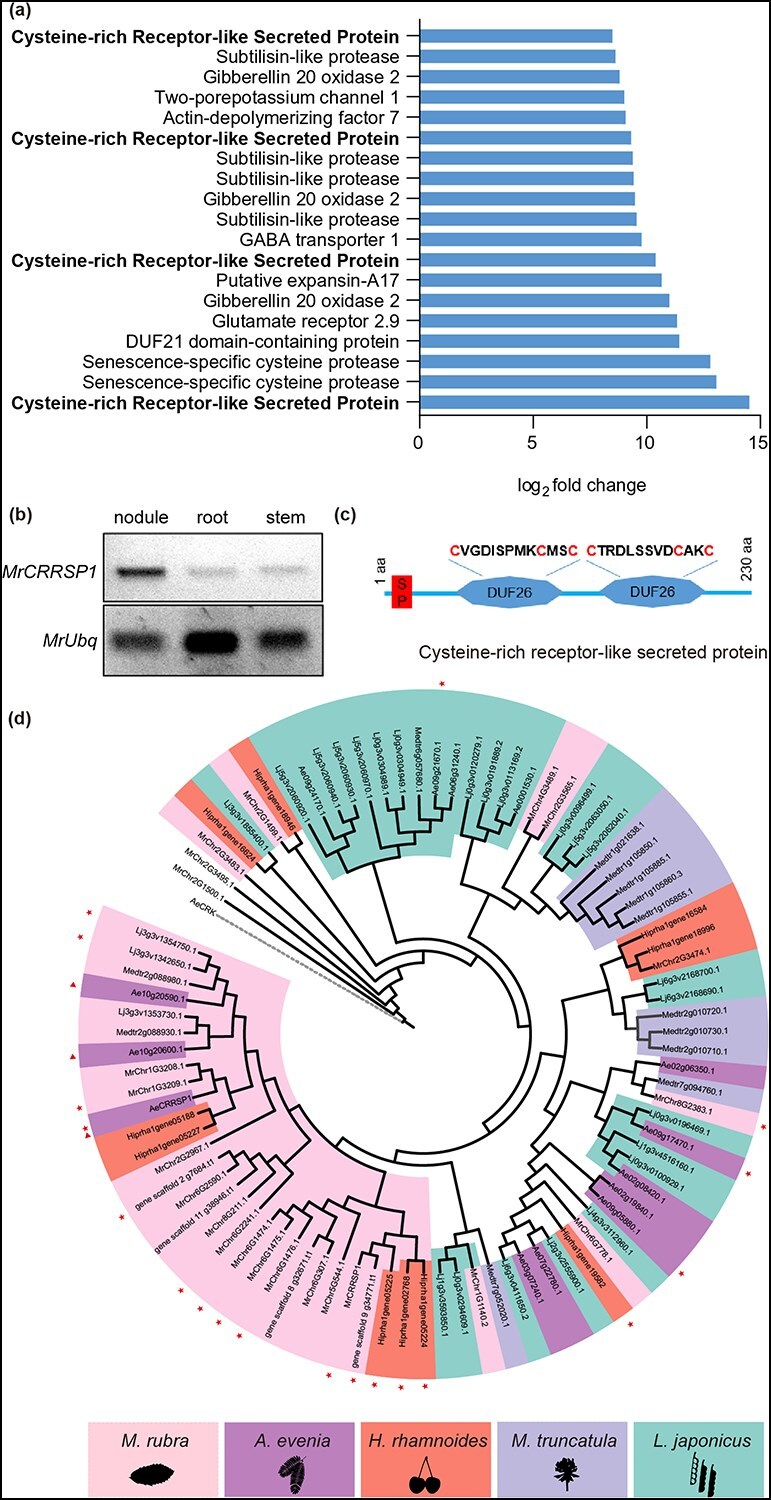
Characterization of MrCRRSP1 in *M. rubra.* (a) The top 20 upregulated genes within *M. rubra* nodules. (b) Expression levels of *MrCRRSP1* in nodules, roots, and stems. Gene expression was detected using RT-PCR. (c) Schematic representation of the CRRSP protein structure. The cysteine-rich domain (C-6X-C-2X-C) of unknown function (DUF26) motif is illustrated. (d) Phylogenetic tree of CRRSP. The amino acid sequences of CRRSP were employed to construct the phylogenetic tree via the maximum likelihood method. CRK protein sequences served as the outgroup. Asterisks denote expression within nodules and triangles denote the homologs of MrCRRSP1 in *A. evenia*.

We subsequently attempted to identify the genes required for nodulation independent of LCO. Notably, among the top 20 genes exhibiting significant upregulation in the transcriptomes of *M. rubra* nodules, we discovered four genes encoding CRRSPs (Fig. 4a and Table S1). A cysteine-rich receptor-like kinase (CRK) in *Aeschynomene evenia* (AeCRK) is thought to have undergone evolutionary adaptation to facilitate symbiosis with a *Bradyrhizobia* strain that lacks LCO, as disruptions of AeCRK result in a loss of nodulation in both the roots and stems of *A. evenia* [21]. This observation prompted us to investigate whether MrCRRSPs play a role in LCO-independent nodulation. The gene with the highest expression level was chosen for further studies and designated as *MrCRRSP1*. RT-PCR confirmed elevated *MrCRRSP1* expression within the nodules (Fig. 4b).

To ascertain whether MrCRRSP1 is phylogenetically correlated with LCO-independent nodulation, we identified all CRRSPs from the representative LCO-dependent legume species *M. truncatula* and *L. japonicus*, as well as the LCO-independent legume species *A. evenia*, and the LCO-independent actinorhizal species *Hippophae rhamnoides*. This identification was based on a predicted sequence comprising a signal peptide followed by two cysteine-rich domains of unknown function (DUF26) (Fig. 4c). Phylogenetic analysis revealed that CRRSPs from various species clustered together, albeit with some exceptions (Fig. 4d). Notably, over 80% of *CRRSP* genes in *M. rubra* (9/11) and *H. rhamnoides* (4/5) and 31% of those in *A. evenia* (4/13) were expressed in the nodules (Fig. 4d and Table S3). Conversely, <15% of *CRRSP* genes in *M. truncatula* (0/6) and *L. japonicus* (2/15) were expressed in the nodules (Fig. 4d and Table S3). These findings support the notion that the emergence of *CRRSP* genes may be associated with LCO-independent nodulation.

Because some CRRSPs possess antifungal properties [22], we hypothesized that MrCRRSP1 might serve to defend against harmful pathogens, thereby enabling symbiotic *Frankia* to occupy advantageous ecological niches. To test this hypothesis, we purified prokaryotic MrCRRSP1 proteins and conducted *in vitro* antifungal assays [23]. Compared with glutathione-S-transferase (GST) alone, GST-tagged MrCRRSP1 proteins did not significantly impede the mycelial growth of *Fusarium oxysporum* and *Magnaporthe oryzae*, two pathogenic fungi (Fig. S4). This suggests that MrCRRSP1 does not exhibit antifungal activity, but rather may play a role in the interaction between nitrogen-fixing bacteria and their host organisms.

### Overexpression of *MrCRRSP1* in *A. indica* promotes nodule formation

**Figure 5:**
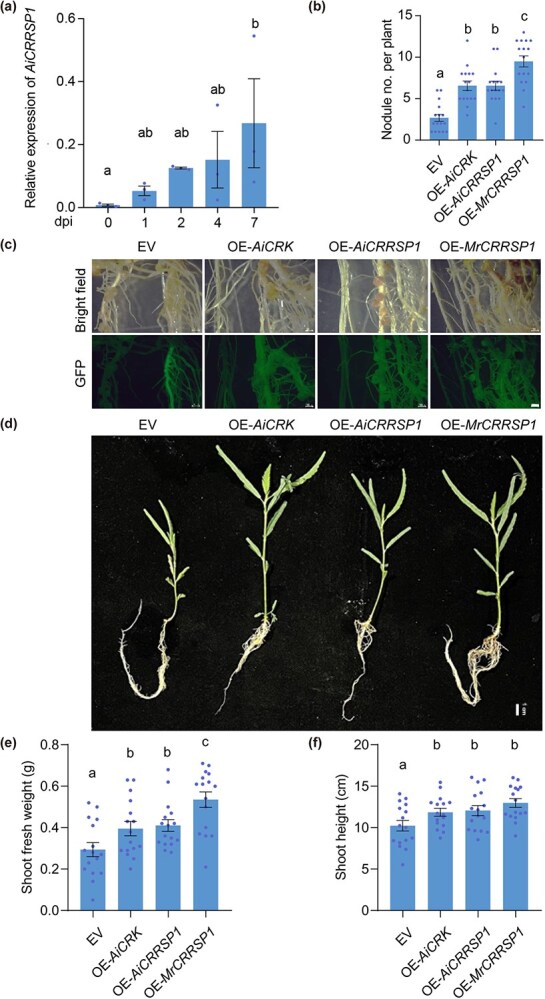
OE of *CRRSP1* in *A. indica* promotes nodule formation. (a) The transcript levels of *AiCRRSP1* in *A. indica* roots following inoculation with *Bradyrhizobium* sp. ORS285. The transcript levels were detected by RT-qPCR. Data are means ± SE (*n* = 3). Different letters above the bars indicate significant differences between different groups (*P* ≤ 0.05, one-way ANOVA). (b–f) OE of *AiCRK, AiCRRSP1,* and *MrCRRSP1* in *A. indica* via hairy root transformation. Images were captured 3 wpi with *Bradyrhizobium* sp. ORS285. Representative images are shown in (c, d). Fluorescence signals represent transgenic roots expressing fluorescent proteins in (c). EV, empty vector; Bars, 1000 μm in (c) and 1 cm in (d). The number of nodules per plant (b), plant growth including shoot fresh weight (e), and shoot height (f) were measured at 3 wpi. Data are means ± SE (*n* = 16). Different letters above the bars indicate significant differences between different groups (*P* ≤ 0.05, one-way ANOVA).

To explore the role of MrCRRSP1 in nodulation, we utilized the LCO-independent legume species *A. indica*. Given the unavailability of the whole-genome sequence of *A. indica*, we initially identified the three orthologs of MrCRRSP1 in *A. evenia*, and the homolog of Ae10g20570.1 in *A. indica* was designated as AiCRRSP1 (Fig. 4d and Fig. S5). The transcript levels of *AiCRRSP1* were significantly increased in the roots in the early stage following inoculation with *Bradyrhizobium* sp. ORS285, a strain lacking NodABC (Fig. 5a). Through *A. rhizogene*-mediated hairy root transformation, *MrCRRSP1*, *AiCRRSP1*, and *AiCRK* were transiently overexpressed in *A. indica* seedlings (Fig. 5b and c), and the number of nodules was quantified at 3 wpi with ORS285. In contrast to OE of an empty vector, the OE of *MrCRRSP1*, *AiCRRSP1*, and *AiCRK* led to a significant increase in the number of nodules (Fig. 5b and c). This, in turn, facilitated seedling growth, as evidenced by increased plant height and enhanced biomass (Fig. 5d–f).

However, the OE of *MrCRRSP1* in *L. japonicus* and *Medicago sativa* did not result in an increase in nodule number or plant biomass (Fig. S6). Furthermore, we transiently silenced the *AiCRRSP1* and *AiCRK* genes in *A. indica* seedlings. Silencing *AiCRK* significantly inhibited nodule formation and plant growth, whereas silencing *AiCRRSP1* only modestly decreased the nodule number, suggesting that *CRRSP1* might function redundantly or have undergone subfunctionalization in *A. indica* (Fig. S7). Nevertheless, the OE of *MrCRRSP1* and *AiCRRSP1* in *A. indica* enhanced nodulation and seedling growth.

### MrCRRSP1 promotes ORS285 enrichment in lateral root cracks

**Figure 6:**
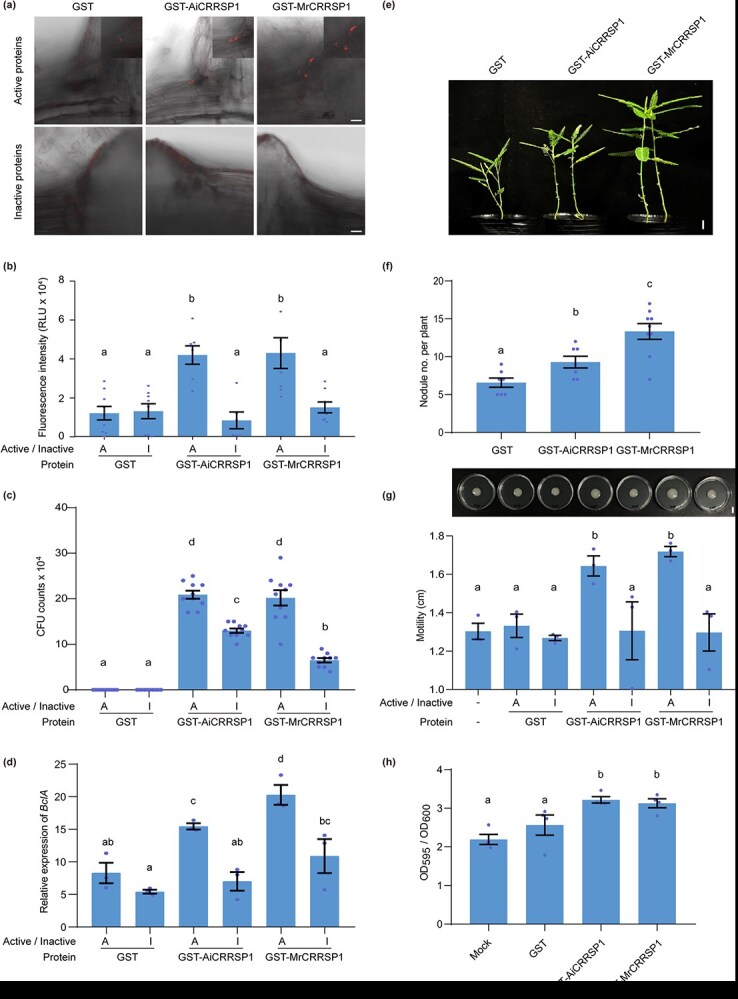
CRRSP1 promotes the enrichment of *Bradyrhizobium* sp. ORS285 in lateral root cracks. (a) The detection of mCherry-labeled *Bradyrhizobium* sp. ORS285 in lateral root cracks. GST-tagged MrCRRSP1 and AiCRRSP1 proteins were affinity purified. Inactive proteins were prepared by heating. *Aeschynomene indica* roots were treated with the purified proteins and subsequently inoculated with ORS285-mCherry. Fluorescence was observed at 1 day postinoculation (dpi). Bars, 20 μm. (b) Quantification of fluorescence intensity in (a). (c) Detection of bacterial levels in roots by CFU assay. (d) Detection of bacterial levels in roots by qPCR assay. The relative expression levels of bacterial *BclA* gene were normalized to *A. indica Elongation Factor 1* (*AiEF1*). (e) Representative image of plant growth at 21 dpi. Bars, 1 cm. (f) Quantification of nodule number per plant at 21 dpi. (g) Effect of MrCRRSP1 proteins on ORS285 motility. (h) Effect of MrCRRSP1 proteins on ORS285 biofilm. Data are means ± SE (*n* = 10 in b, c, and f; 3 in d; 4 in g and h). Different letters above the bars indicate significant differences between different groups (*P* ≤ 0.05, one-way ANOVA).

To determine whether MrCRRSP1 plays a role in the early stages of bacterial entry, ORS285 was labeled with red fluorescent protein (mCherry) by incorporating an mCherry-containing plasmid to facilitate bacterial observation (Fig. S8). Purified prokaryotic GST, GST-MrCRRSP1, and GST-AiCRRSP1 proteins (Fig. S9) were sprayed onto the roots, followed by inoculation with ORS285-mCherry. Compared to GST alone, GST-MrCRRSP1 and GST-AiCRRSP1 enhanced the accumulation of ORS285-mCherry in lateral root cracks, whereas this phenomenon was diminished when proteins were inactivated by heating (Fig. 6a and b). Colony counting and qPCR analysis confirmed a higher bacterial load in roots treated with GST-MrCRRSP1 and GST-AiCRRSP1 compared to those supplemented with GST alone (Fig. 6c and d). These findings suggest that MrCRRSP1 and AiCRRSP1 may play roles in bacterial attachment. Therefore, we investigated the effect of MrCRRSP1 and AiCRRSP1 on bacterial motility and biofilm formation. Our results indicated that the addition of MrCRRSP1 and AiCRRSP1 significantly promoted ORS285 motility and biofilm formation. Collectively, these findings suggest that CRRSP1 promotes bacterial attachment to lateral root cracks to enhance intercellular infection.

## Discussion

**Figure 7:**
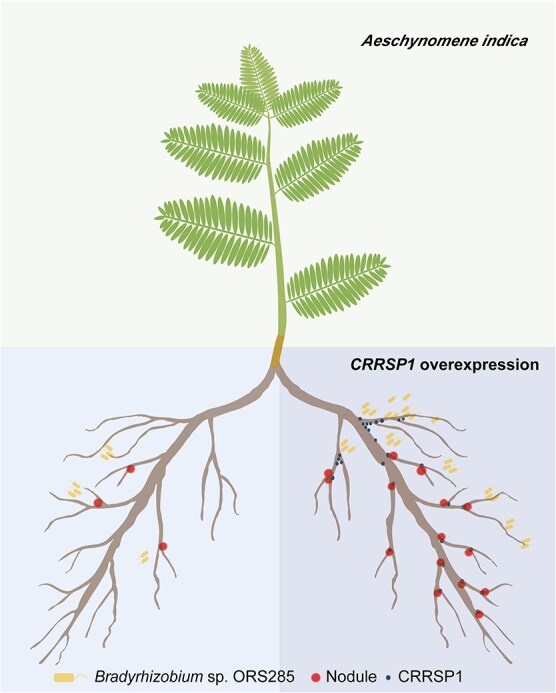
Schematic model illustrating the role of CRRSP1 in facilitating *Bradyrhizobium* sp. ORS285 attachment to *A. indica* lateral root cracks, which promotes intercellular infection and enhances nodulation. . The model was created using Bio-Render (https://BioRender.com).

The interaction between *M. rubra* and *Frankia* represents an ancient type of symbiosis that offers an exemplary system for elucidating the evolution of nodule symbiosis. In this study, we identified a *CRRSP1* gene that was substantially upregulated in the nodules of *M. rubra*. The exogenous application of CRRSP1 proteins to *A. indica* significantly increased rhizobial adherence to cracks in the lateral root base, which serves as an intercellular entry route, thereby enhancing nodulation (Fig. 7). These results provide valuable insights into the molecular mechanisms underlying intercellular infections.

N-fixing bacteria establish symbiotic relationships with their host plants via two entry mechanisms [12]. Most leguminous plants colonize using the root hair curl method, whereas ~25% of legumes utilize intercellular entry mechanisms [24, 25]. Compared to abundant literature on the mechanism of root hair infection, few studies have explored the genes associated with the intercellular infection pathway [12]. Actinorhizal plants in the orders Rosales and Cucurbitales are infected by *Frankia* via the intercellular route, whereas most actinorhizal plants within the order Fagales are infected via the root hair curl method [26, 27]. *Myrica rubra* belongs to the order Fagales; however, the *Frankia* class, which interacts with *M. rubra*, lacks *Nod* genes [28]. Furthermore, we found that *M. rubra* lacks orthologs of Nod factor receptors. Collectively, these findings suggest that *M. rubra* may be infected via an intercellular entry mechanism, despite belonging to the order Fagales.

CRRSP and CRKs are members of a family characterized by the presence of DUF26 domains [29]. A subset of CRRSPs, each with one or two DUF26 domains, may have evolved from CRKs following multiple rounds of duplication and subsequent loss of the kinase domain [29]. CRKs play important roles in plant immunity, including pattern- and effector-triggered immunity [30]. Some CRKs interact with pattern recognition receptors and serve as potential components of receptor complexes. For example, CRK28 partners flagellin receptors to modulate the production of reactive oxygen species in response to flagellin [31]. Numerous CRKs are capable of directly influencing downstream immune responses, such as Ca^2+^ influx, reactive oxygen species generation, mitogen-activated protein kinase activation, and callose deposition [30]. Additionally, DUF26 domains can bind to sugar residues in the fungal cell wall, which can inhibit fungal growth. Examples include CRRSP from *Ginkgo biloba*, known as Ginkbilobin2, which inhibits the growth of *Botrytis cinerea*, *F. oxysporum*, and *F. culmorum* [22]; maize CRRSPs, known as antifungal proteins 1 and 2, which target *Ustilago maydis* [32]; and wheat TaCRR1, which is effective against *Rhizoctonia cerealis* [33]. Our findings suggest that MrCRRSP1 does not display antifungal activity against *B. cinerea* or *F. oxysporum*; however, further investigation is needed to ascertain whether MrCRRSP1 inhibits other fungal species.

DUF26 domain-containing proteins also contribute to symbiosis. A mutation in a *CRK* gene of *M. truncatula*, referred to as SymCRK, impedes nodule development and bacteroid differentiation, resulting in necrotic nodules characterized by an increased accumulation of phenolic compounds and expression of defense-related genes [34]. Therefore, SymCRK in *M. truncatula* may regulate nodule symbiosis by suppressing plant immune responses. Furthermore, a *CRK* mutation in *A. evenia* disrupts its symbiotic relationship with ORS278, a photosynthetic *Bradyrhizobia* strain that lacks *Nod* genes and employs an intercellular infection method [35]. Because *Aecrk* mutants exhibit a complete absence of stem and root nodules, AeCRK is hypothesized to play a role in the early stages of Nod-independent intercellular entry [35]. Additionally, orthologs of *AeCRK* are found in other legumes that use intercellular infection, but not in those that use the root hair infection process, supporting the hypothesis that AeCRK plays a role in the intercellular infection route [35]. In this study, we observed that MrCRRSP1 and AiCRRSP1 promoted bacterial motility and recruited bacteria toward the lateral root base. Although CRRSP1 is unlikely to function as the chemotactic reagent because it was evenly sprayed on the roots, this protein may facilitate bacterial attachment. Given that both AeCRK and CRRSP1 contain DUF26 domains, future studies should investigate whether the function of CRRSP1 in intercellular infections is related to AeCRK. Furthermore, given the high expression of CRRSP1 in mature nodules, it would be valuable to explore its potential role in nodule maturation and function. However, the precise molecular mechanisms underlying CRRSP1-mediated intercellular infection and nodule development remain to be elucidated and warrant further investigation.

In summary, we identified a CRRSP1 peptide that was significantly upregulated in the nodules of *M. rubra*. The application of purified CRRSP1 to *A. indica* enhanced the bacterial colonization of lateral root base cracks. This suggests that the CRRSP1 protein holds potential for enhancing nodulation through an intercellular infection mechanism.

## Materials and methods

### Plant materials and growth conditions

*Myrica rubra* seedlings were obtained from Lanxi District, Jinhua City, Zhejiang Province, China. *Aeschynomene indica* seeds were obtained from Longyou County Farmers’ Market, Quzhou City, Zhejiang Province, China. *Lotus japonicus* wild type (MG20) and *nfr5* mutants of *L. japonicus* were kindly provided by Professor Yangrong Cao of Huazhong Agricultural University, China. The seeds were germinated as previously described [36]. Briefly, seeds were immersed in 95% concentrated sulfuric acid for 10 min. After washing three times with sterile water, the seeds were incubated with 10% sodium hypochlorite for 10 min and rinsed with sterile water seven times. Subsequently, seeds were incubated in water overnight at 4°C. For the seedling assay of *L. japonicus* and *A. indica*, seeds were germinated on agar plates with modified Buffered Nod Medium (BNM) [37]. Seedlings were grown in a growth chamber at 24°C and 75% relative humidity with a 12-h photoperiod.

### Bacterial strains and culture conditions

*Bradyrhizobium* sp. ORS285 [38], DsRed-labeled *Mesorhizobium loti* [39] and *Sinorhizobium meliloti* 2011 [37] were cultured on Yeast Mannitol Agar (10 g/l Mannitol, 0.25 g/l K_2_HPO_4_, 0.25 g/l KH_2_PO_4_, 0.2 g/l MgSO_4_·7H_2_O, 0.1 g/l NaCl, 3 g/l Yeast Extract, 15 g/l Agar, pH 6.8–7.0) and stored in 25% glycerol at −80°C. *Agrobacterium rhizogenes* AR1193 [40] was cultured on Luria–Bertani medium. The mCherry-labeled *Bradyrhizobium* sp. ORS285 was generated using a previously constructed plasmid, pBRM-mCherry [41]. Briefly, the pBRM-mCherry plasmid was transferred into the competent *Bradyrhizobium* sp. ORS285 cells by electroporation method.

### RNA isolation and RT-qPCR

Total RNA was extracted using an Easy Plant RNA Extraction Kit (DR0407050; Easy-Do Biotech, Hangzhou, China). Reverse transcription reactions were performed using 1 μg of total RNA with HiScript II Q RT SuperMix for qPCR with gDNA wiper (R223–01, Vazyme Biotech, Nanjing, China). SYBR Green Master Mix was used for the RT-qPCR reactions (Vazyme Biotech). Data were analyzed with the 2^−ΔΔCT^ (cycle threshold) method [42] and normalized to the expression of the reference genes: *UBIQUITIN* for *M. rubra* and *ELONGATION FACTOR1* (*EF1*) for *A. indica*. The primers used for RT-qPCR are listed in Table S4.

**Table S4:** 

### Plasmid construction

The primers used for gene cloning are listed in Table S4. The fragments of interest were cloned into a pDONR-Zeo plasmid via BP cloning then cloned into the pUB-GFP vector via LR cloning to obtain the following constructs: *p35S::GFP-pLjUbq1::LjNFR5* (*OE-LjNFR5*), *p35S::GFP-pLjUbq1::MrChr3G3944.t1* (*OE-MrNFP*), *p35S::GFP-pLjUbq1::AiCRK* (*OE-AiCRK*), *p35S::GFP-pLjUbq1::AiCRRSP1* (*OE-AiCRRSP1*), and *p35S::GFP-pLjUbq1::MrCRRSP1* (*OE-MrCRRSP1*). The pUB-GWS-GFP vector was used to construct the *p35S::GFP-pLjUbq1::AiCRK-RNAi* (*AiCRK RNAi*) and *p35S::GFP-pLjUbq1::AiCRRSP1-RNAi* (*AiCRRSP1 RNAi*) vectors. All constructs were introduced into *A. rhizogenes* AR1193 cells for hairy root transformation.

### Hairy root transformation

Hairy root transformation was performed as described previously [40]. AR1193 cells harboring each construct, or its empty vector, were streaked on an Luria–Bertani medium plate with appropriate antibiotics for 2 days at 28°C. Then, sterilized 4-day-old seedlings were cut in the middle of the hypocotyl and incubated in bacterial suspension culture for 10 min. Seedlings with cotyledons were transferred onto BNM in a growth chamber (25°C in the dark for the first 3 days, then 24°C with a 16-h light/8-h dark cycle for the next 7 days). Next, the plants were transferred onto fresh medium containing 100 μg/ml cefotaxime sodium and grown for 10 days in a growth chamber (24°C; 16-h light/8-h dark cycle). Transgenic hairy roots were identified by GFP fluorescence under a fluorescence stereoscopic microscope (SMZ18; Nikon, Tokyo, Japan) and used for further experiments.

### Nodulation analysis

For the plate experiments, seedlings germinated on 0.8% agar plates were transferred to the BNM agar medium. The 7-day-old seedlings were inoculated with ~2 × 10^7^ cfu/ml (OD_600_ = 0.01) bacteria per plant. The number of nodules per plant was analyzed at 3–4 wpi. All results were confirmed by separate biological experiments.

For soil experiments, germinated seedlings or hair-root-transferred seedlings were transferred to pots (8 × 8 × 8 cm) filled with a vermiculite: perlite matrix in a ratio of 3:1. After 3 days, plants were inoculated with 2 × 10^7^ cfu/ml (OD_600_ = 0.01) bacteria per plant. BNM liquid medium was added to each pot at intervals of 3 days. The number of nodules per plant was counted at 3–4 wpi. All results were confirmed by separate biological experiments.

### *Myrica rubra* nodule section and toluidine blue staining

Nodules were harvested, rinsed with 0.1 M phosphate buffer (pH 7.4), and fixed with 1% osmium acid at 24°C in the dark for 7 h. After dehydration, the nodules were embedded using an EMbed 812 Embedding Kit (SPI, West Chester, PA, USA). The semithin sections were stained with toluidine blue. Nodule structures were observed under a light microscope (Eclipse Ni-U; Nikon, Tokyo, Japan).

### RNA-seq analysis

Root and nodule materials were collected from *M. rubra* seedlings in Lanxi District, Jinhua City, Zhejiang Province, China. Total RNA were extracted and quality control of DNase I-treated mRNA was performed with an Agilent 2100 Bioanalyzer. cDNA libraries were generated, and sequencing was performed using the Illumina HiSeq 2500 platform according to the manufacturer’s instruction. Raw data (raw reads) of fastq format were firstly processed through fastp software. All the downstream analyses were based on the clean data with high quality. For data analysis, sequence reads were firstly aligned to the *M. rubra* genome [17], adjusting the minimum and maximum intron lengths to 20 and 4000 bp, respectively. Second, the mapped reads were assembled using Cufflinks (Galaxy v.2.2.1.3) with default settings [43]. Third, to identify DEGs, the assembled transcripts from three independent biological replicates of nodules and roots were combined and compared using Cuffmerge (Galaxy v.2.2.1.2) with default settings [43]. Genes with an expression change of at least 2-fold (false discovery rate < 0.05, *P* < 0.05) were considered differentially expressed and used for downstream analysis.

### Sequence alignment and phylogeny analysis

For phylogenetic analysis of the LYK and CRRSP family, amino acid sequences and the expression atlas were obtained from MtExpress (https://medicago.toulouse.inrae.fr/mtexpress), *Aeschynomene* Base (https://aeschynomenebase.fr), *Lotus* Base (http://lotus.au.dk), *M. rubra* Database (http://www.bayberrybase.cn), NCBI (http://www.ncbi.nlm.nih.gov/), and CNGB Nucleotide Sequence Archive (https://db.cngb.org/cnsa). Protein sequences were subjected to multiple sequence alignments by MAFFT (v.7.310) [44] and phylogenetic tree inference using maximum likelihood/rapid bootstrapping run on CIPRES (http://www.phylo.org) or MEGA X [45]. The tree was visualized using iTOL (https://itol.embl.de/) [46].

### Protein expression and purification

For fusion with the GST tag, the target fragment was inserted into the pDEST15 vector, and the resulting vectors were introduced into *Escherichia coli* BL21 cells [47]. When the cellular density reached an OD_600_ of 0.6, isopropyl β-d-1-thiogalactopyranoside was added at a final concentration of 0.5 mM to induce protein expression at 16°C and 220 rpm for 12 h. The recombinant proteins were purified using glutathione affinity resin then filtered through a sterile 0.22-μm membrane to obtain sterile protein solution. Purified proteins with a final concentration of 100 nmol/l were applied exogenously to *A. indica* seedlings grown on BNM agar plates.

### Detection of mCherry-labeled Bradyrhizobium sp. ORS285

Red fluorescence was observed under a confocal laser-scanning microscope microscope (FV3000; Olympus, Tokyo, Japan). The fluorescence intensity was analyzed with ImageJ software (https://github.com/imagej/ImageJ) as previously described [48]. Briefly, a circular region of interest (ROI) was selected, encompassing the target fluorescence signal, with an area of 3000 pixels. For background correction, 3–5 matched circular ROIs devoid of fluorescence were chosen. The fluorescence intensity was calculated as: integrated density − (area of ROI × mean fluorescence of background readings).

### Colony counting and qPCR analysis

For colony forming unit (CFU) assays, *A. indica* roots were treated with the purified proteins and subsequently inoculated with ORS285-mCherry at 2 dpi. Subsequently, the root surfaces were thoroughly sterilized. Equal weights of root segments were cut and homogenized using a grinder. The homogenate was then serially diluted and plated onto YMA medium supplemented with the appropriate antibiotic. CFUs were enumerated 2 days later. For qPCR analysis, total genomic DNA encompassing both plant and bacterial fractions was isolated from surface-sterilized root samples. The ORS285-specific primer *BclA* was used [49], and *A. indica Elongation Factor 1* was selected as the reference gene.

### Motility assay and biofilm formation assay

The swimming assay was performed following a previously described method [50]. Briefly, *Bradyrhizobium* sp. ORS285 strains were cultured in YMA medium until the OD_600_ reached 0.8. Afterward, the cultures were inoculated on Petri dishes containing semisolid YMA medium with 0.5% (w/v) agar using sterilized toothpicks. The Petri dishes were incubated at 28°C for 48 h to allow the bacterial cells to swim. The diameter of bacterial cells was then measured using the crosshair method with a Vernier caliper. Four replicates were included for each strain. The biofilm formation assay was performed as previously described method [51]. Briefly, *Bradyrhizobium* sp. ORS285 strains were cultured in YMA medium until the OD_600_ reached 0.6. Then, 100 μl of bacterial suspension was aliquoted into a 96-well plate. The plate was incubated statically at 28°C for 2 days, and the optical density was measured at 600 nm using a multimode plate reader system, FlexStation 3 (Molecular Devices). Subsequently, the bacterial suspension was carefully discarded, and the plate was left undisturbed for 5–6 h to allow biofilm stabilization at the bottom. The wells were then gently washed twice with deionized water and air-dried in a fume hood. Next, 125 μl of 0.1% crystal violet solution was added to each well and incubated at room temperature for 30 min. Unbound crystal violet was carefully removed, and the plate was again left undisturbed for 5–6 h to ensure biofilm adherence. After washing two to three times with deionized water and air-drying in the fume hood, the crystal violet dye bound to adherent cells was released by adding 160 ml 33% acetic acid and the optical density was measured at 595 nm, using the same multimode plate reader system.

### Statistical analysis

Data for quantification analyses are presented as means ± standard error (SE) using GraphPad 8. Statistical analyses with Student’s *t*-test or one-way analysis of variance (ANOVA) followed by Duncan’s new multiple range test were performed using SPSS 26. The detailed statistical information and the number of replicates are shown in the figure legends.

### Accession numbers

*Myrica rubra* and *A. indica* sequence data from this article can be found in the GenBank/EMBL data libraries under the following accession numbers: MrCRRSP1, PV055699; AiCRRSP1, PV055700; AiCRK, PV055701; MrNFP, PV055702.

## Supplementary Material

Web_Material_uhaf185

## Data Availability

All data are included in the main figures and supplemental figures. Any additional information required to reanalyze the data reported in this article is available from the corresponding author upon request.
