# Supplementary material for: Cysteine-rich receptor-like secreted protein 1 promotes intercellular infection and enhances nodulation in Aeschynomene indica
Source: Hortic Res. 2025 Jul 22;12(10):uhaf185. doi: 10.1093/hr/uhaf185 (PMC12537019; doi:10.1093/hr/uhaf185)
Supplement: Web_Material_uhaf185 [file web_material_uhaf185.zip › 3_Huang_CRRSP_suppl_06272025-huang.pdf]

Cysteine-rich receptor-like secreted protein 1 promotes intercellular infection and enhances nodulation in *Aeschynomene indica*

Zeming Huang[1], Guiling Ren[1], Xijie Guo[1], Yaxing Su[1], Yuchen Wang[1], Shuwen Zhang[2], Xingjiang Qi[2], Huijie Lu[3], Jiazhang Lian[4], Yan Liang[1*]

The supplementary file includes Figures S1-S7 and Table S4.

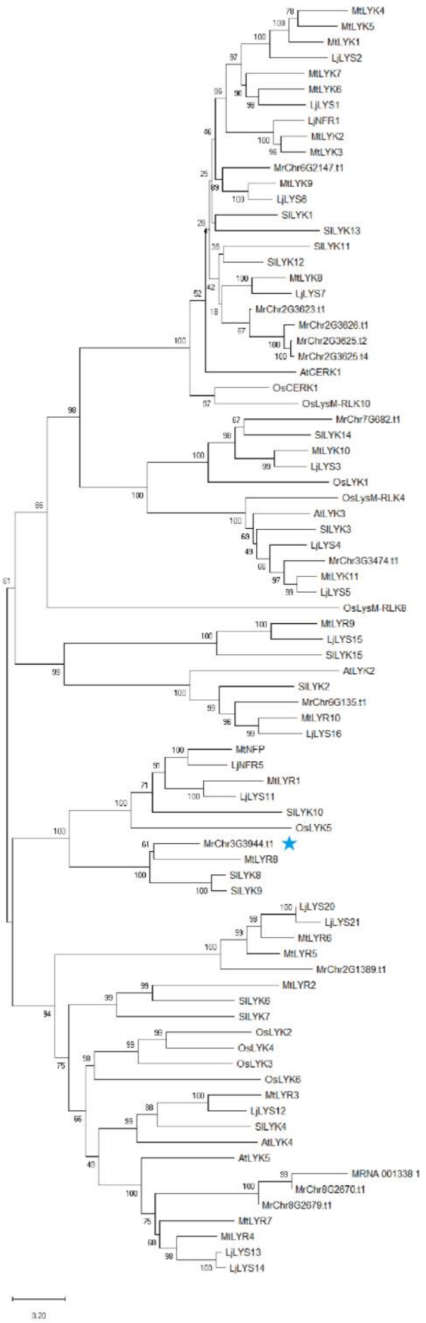

Figure S1. Phylogenetic tree of the NFP family

An unrooted phylogenetic tree of NFP protein homologs was constructed using the neighbor-joining method. Species of origin: *At*, *Arabidopsis thaliana*; *Mt*, *Medicago truncatula*; *Lj*, *Lotus japonicus*; *Sl*, *Solanum lycopersicum*; *Os*, *Oryza sativa*. Branches were labeled with their respective bootstrap values. Asterisk denotes *MrNFP*.

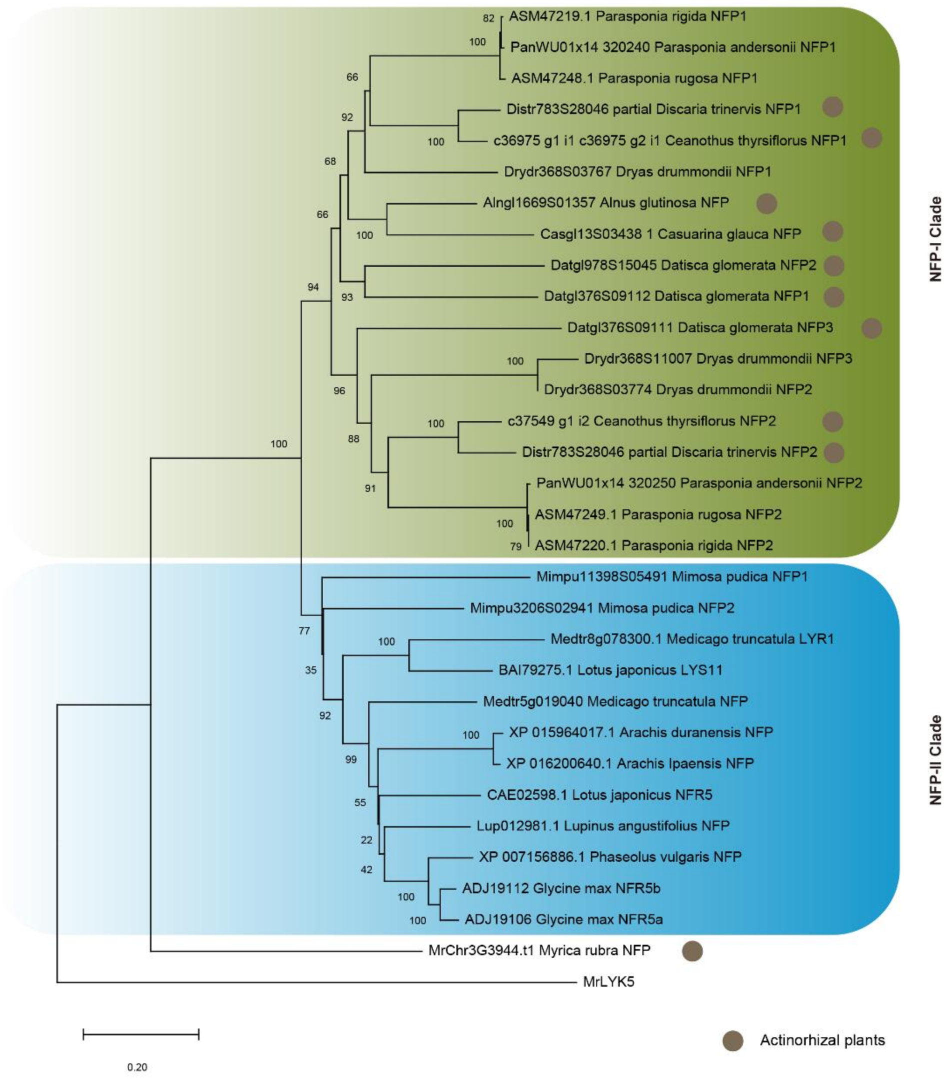

Figure S2. MrNFP is not grouped within the NFP clade

Neighbor-joining phylogenetic reconstruction of the LYR-IA orthogroup, which contains known legume LCO receptors, based on 32 amino acid sequences. Percentage of replicate trees in which the associated taxa clustered together in the bootstrap test (1,000 replicates) is shown next to the branches. Gene duplication was identified in the nitrogen-fixing clade, resulting in two subclades: NFP-I (green) and NFP-II (blue). Evolutionary analyses were conducted using MEGA X. Accession numbers can be found in Rutten et al. (2020) [1].

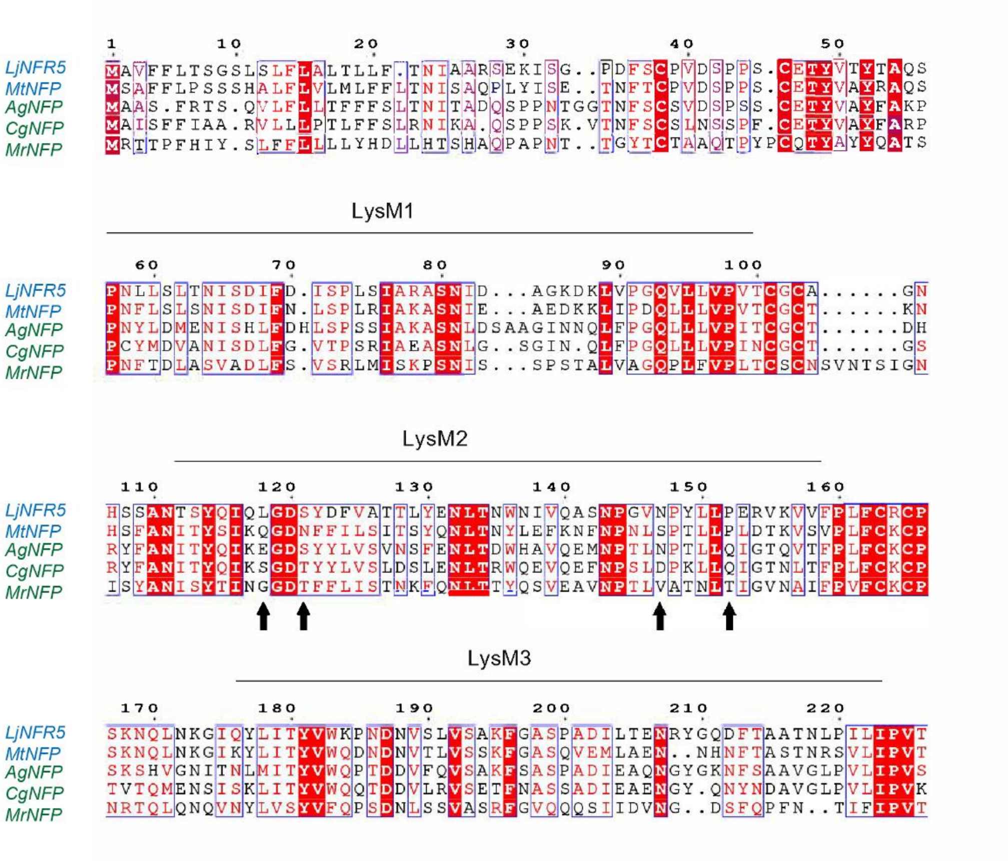

Figure S3. Sequence alignment of the LysM region of MrNFP homologs

Abbreviation: *Lj, Lotus japonicus; Mt, Medicago truncatula; Ag, Alnus glutinosa; Cg, Casuarina glauca*.

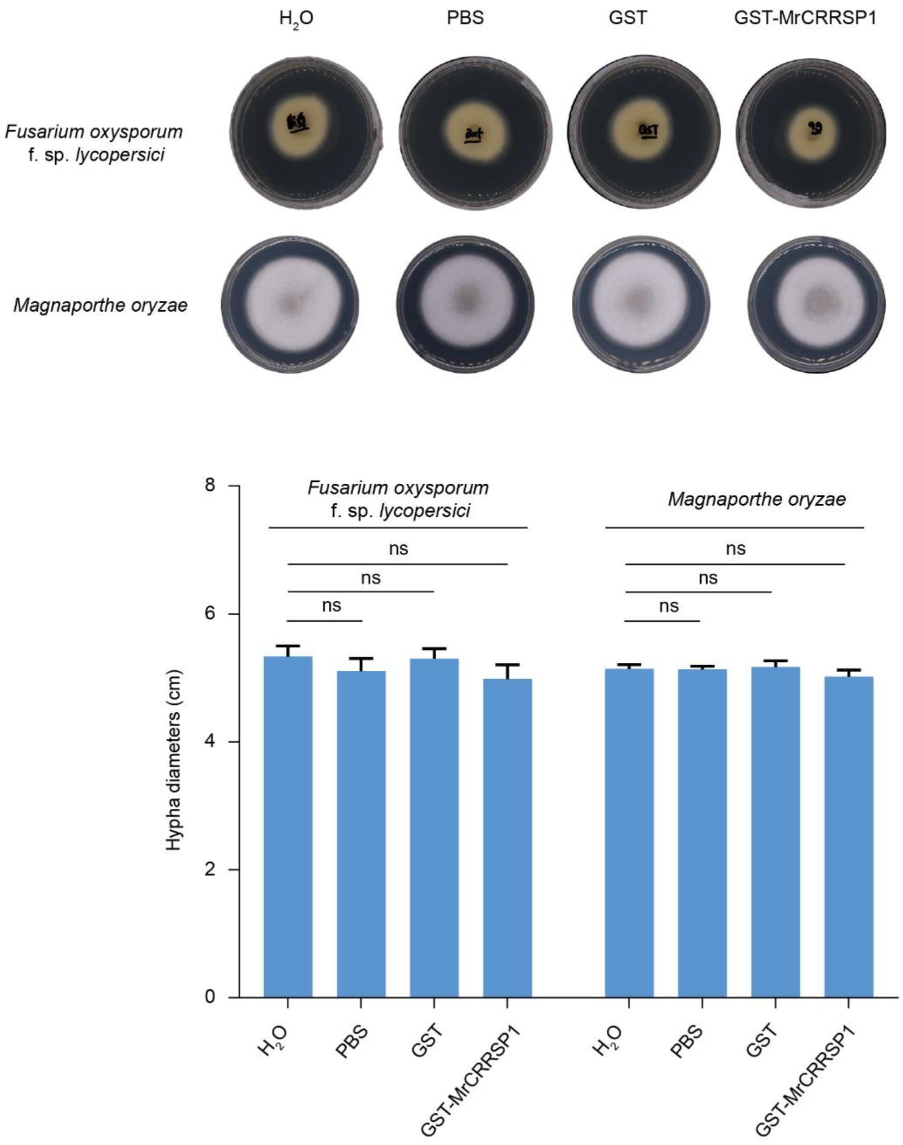

Figure S4. GST-tagged MrCRRSP1 proteins do not significantly impede fungal mycelial growth

Fusarium oxysporum f. sp. *lycopersici (Fol)* hypha diameters (cm) were measured at 4 dpi; *Magnaporthe oryzae* (*Mo*) B157 hypha diameters (cm) were measured at 7 dpi. Data are means ± SE (n = 4). ns, no significant difference ($P \leqslant 0.01$, Student's t-test).

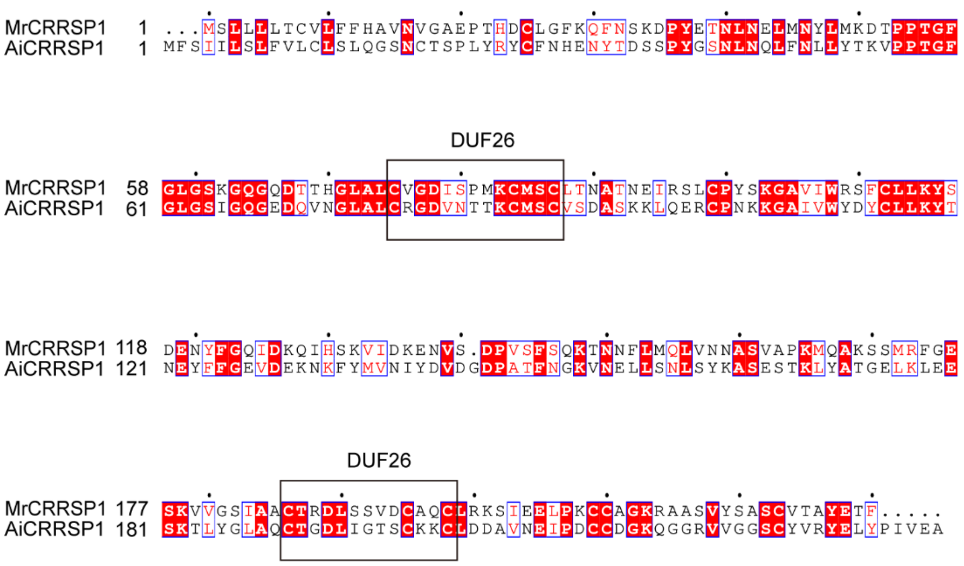

Figure S5. Sequence alignment of MrCRRSP1 and AiCRRSP1

Abbreviations: *Mr*, *Myrica rubra*; *Ai*, *Aeschynomene indica*. Black boxes indicate cysteine-rich domains of unknown function (DUF26).

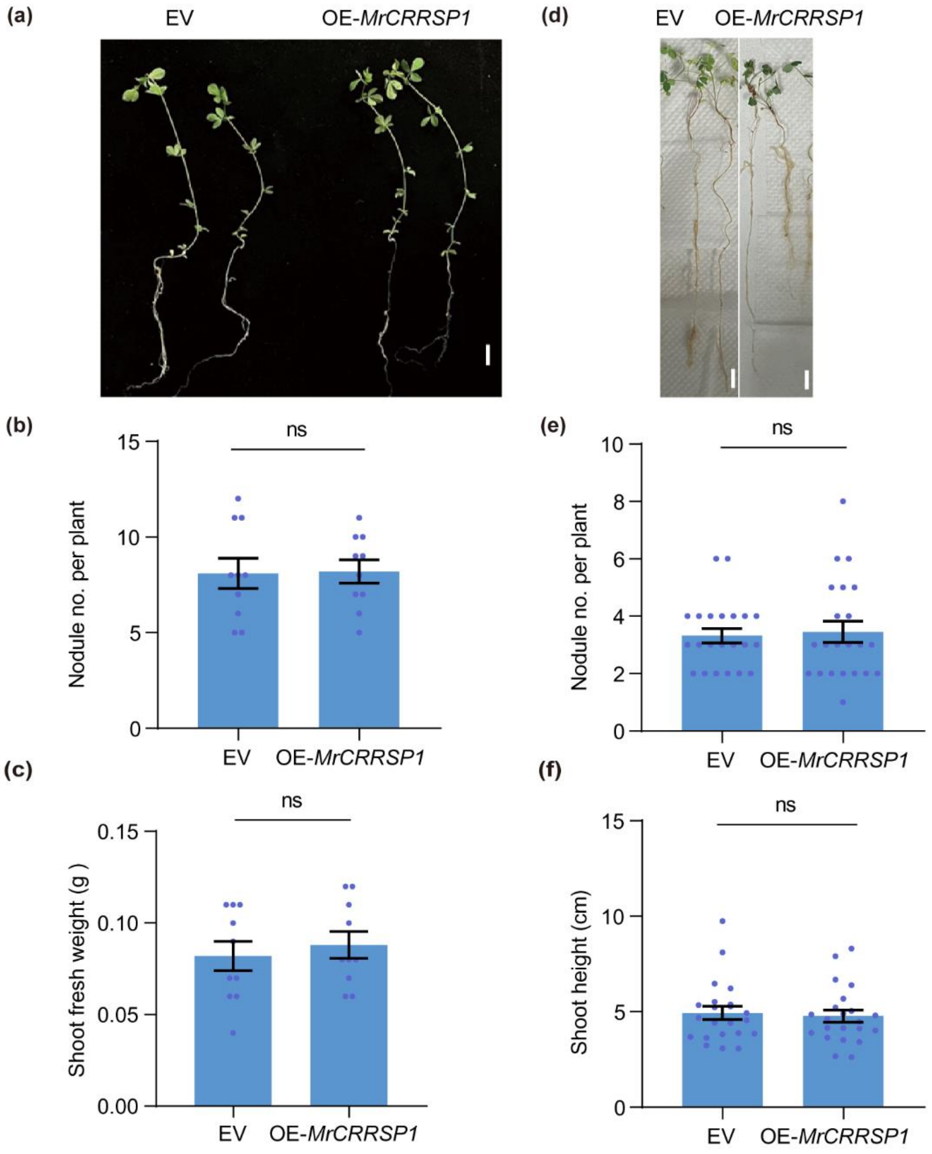

Figure S6. Overexpression of *MrCRRSP1* in *Lotus japonicus* and *Medicago sativa*

(a–c) Overexpression (OE) of *MrCRRSP1* in *L. japonicus* via hairy root transformation. Images were captured 4 wpi with *Mesorhizobium loti*. Representative images are shown in (a). EV, empty vector; bar, 1 cm. Number of nodules per plant (b) and shoot fresh weight (c) were measured at 4 wpi. Data are means ± SE (n = 10). ns, no significant difference (Student's t-test).

(d–f) OE of *MrCRRSP1* in *M. sativa* via hairy root transformation. Images were captured 4 wpi with *Sinorhizobium meliloti* 2011. Representative images are shown in

(d). EV, empty vector; bar, 1 cm. Number of nodules per plant (e) and shoot height (f) were measured at 4 wpi. Data are means ± SE (n = 22). ns, no significant difference (P ⩽ 0.01, Student's t-test).

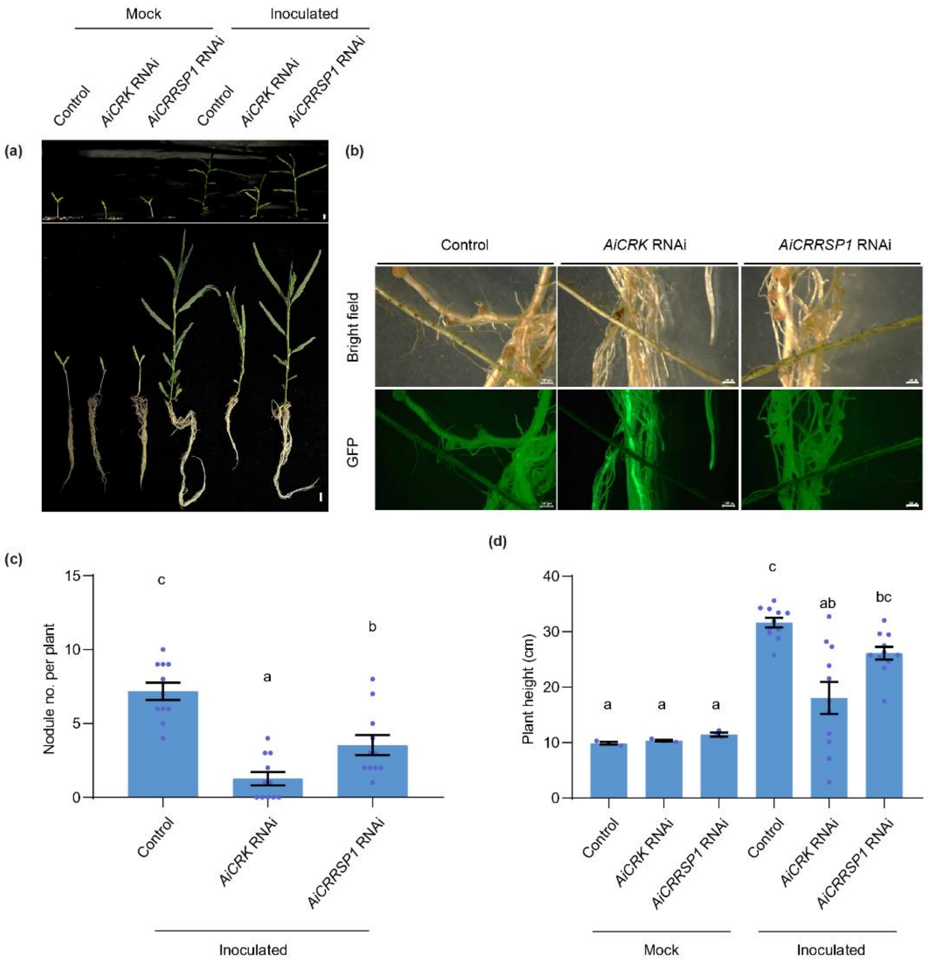

Figure S7. Silencing of *AiCRRSP1* reduces *Aeschynomene indica* nodulation

(a–d) Gene silencing of *AiCRK* and *AiCRRSP1* in *A. indica* via hairy root transformation. Images were captured 4 wpi with *Bradyrhizobium* sp. ORS285. Representative images are shown in (a) and (b). Green fluorescence indicates transgenic roots. EV, empty vector; bars, 1 cm in (a) and 1,000 μm in (b). Number of nodules per plant (c) and plant height (d) were measured at 4 wpi. Data are means ± SE (n = 11). Different letters above the bars indicate significant differences between different groups (P ⩽ 0.05, one-way ANOVA).

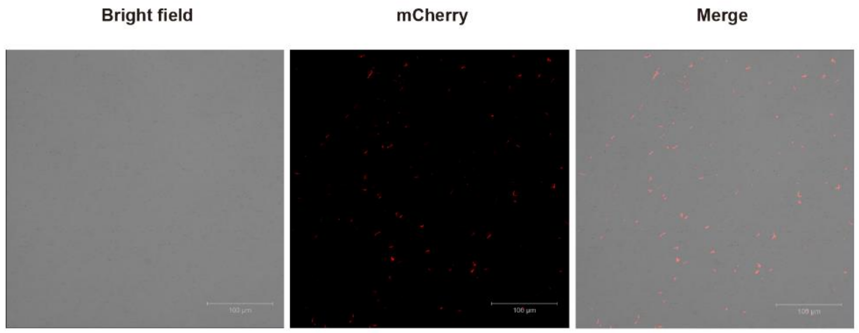

Figure S8. Detection of *Bradyrhizobium* sp. ORS285-mCherry

Red fluorescence from *Bradyrhizobium* sp. ORS285-mCherry detected under a confocal microscope.

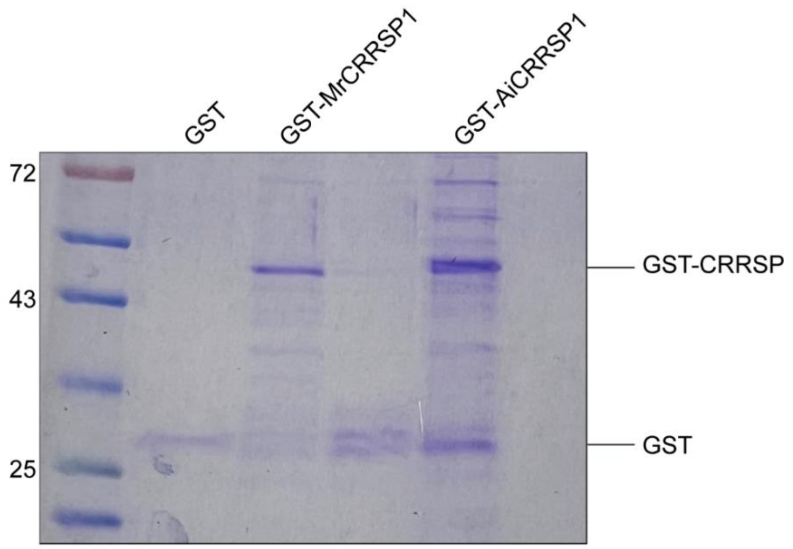

Figure S9. Detection of purified recombinant proteins

MrCRRSP1 and AiCRRSP1 were N-terminally fused to a glutathione-S-transferase (GST) tag and expressed in *E. coli*. Purified recombinant proteins were run on an SDS-PAGE gel and stained with Coomassie Brilliant Blue (CBB).

Table S4 List of primers used in this study

Primer name	Sequences (5' to 3')	Purpose
MrUbq-F	AGCAGAGGCTTATTTTCGCCGG	RT-qPCR
MrUbq-R	GGACCAAGTGGAGGGTGGATTC	
MrNFP-qRT-F	ATGGATGGATGAGGTTCTGATG	
MrNFP-qRT-R	GCCTCTTTGATGGGTCTCTATG	
MrCRRSP1-qRT-F	GTCGGTTCGATAGCTGCG	
MrCRRSP1-qRT-R	GAAGGTTTCGTATGCCGTG	
qRT-LjeIF4A-F	AGAGGGTTTAAAGATCAAAT	
qRT-LjeIF4A-R	ATGTCAATTCATCACGTTTT	
AiEF1-F	TGCTGGTATGGTTAAGATGGTTCC	
AiEF1-R	TTCTTCTTCTGTGCTGCCTTGG	
qRT-AiCRK-F	AAGGCTCTATGGCCTCTTCCT	
qRT-AiCRK-R	AGTGTCTGTGCTTGTGCAAAG	
qRT-AiCRRSP1-F	CCAAAGTTCCTCCTACTGGTTT	
qRT-AiCRRSP1-R	TGTTGACATCACCTCTGCATAA	
BclA-F	AACGCGCTGCAGGAATA	qPCR
BclA-R	GCAGCCACTGATTGAGATAGA	
LjNFR5-GW-F	GGGGACAAGTTTGTACAAAAAAGCAGGCTATGGCTGTCTTCTTTCTTAC	Gene cloning
LjNFR5-GW-R	GGGGACCACTTTGTACAAGAAAGCTGGGTACGTGCAGTAATGGAAGTCAC	
MrNFP-GW-F	GGGGACAAGTTTGTACAAAAAAGCAGGCTATGAGAACCACACCCTTTCACATT	

MrNFP-GW-R	GGGGACCACTTTGTACAAGAAAGCTGGGTTCTTGCCACTACTAGTGGATCGG
MrCRRSP1-GW-F	GGGGACAAGTTTGTACAAAAAAGCAGGCTACATGTCTCTCTTGTTGCTAACCT
MrCRRSP1-GW-R	GGGGACCACTTTGTACAAGAAAGCTGGGTCGAAGGTTTCGTATGCCGTG
AiCRK-GW-F	GGGGACAAGTTTGTACAAAAAAGCAGGCTACATGTATCTCTTCCACAAGAAGTACAG
AiCRK-GW-R	GGGGACCACTTTGTACAAGAAAGCTGGGTCATAGAAAGTAGAAACAGAGCTGG
AiCRRSP1-GW-F	GGGGACAAGTTTGTACAAAAAAGCAGGCTACATGTTCAGCATCATTCTAAGTCTCT
AiCRRSP1-GW-R	GGGGACCACTTTGTACAAGAAAGCTGGGTCGGCCTCAACAATAGGGTACA
RNAi-AiCRK-F	GGGGACAAGTTTGTACAAAAAAGCAGGCTACAGGCTCTATGGCCTCTTC
RNAi-AiCRK-R	GGGGACCACTTTGTACAAGAAAGCTGGGTCCGCCTTGTTCATCAACTGTGA
RNAi-AiCRRSP1-F	GGGGACAAGTTTGTACAAAAAAGCAGGCTACGCGATGTCCAAACAAGAAAG
RNAi-AiCRRSP1-R	GGGGACCACTTTGTACAAGAAAGCTGGGTCGGCCTCAACAATAGGGTACA

A separate Excel file contains the following tables: S1, S2, and S3.

Table S1 Differentially up-regulated and down-regulated genes in *M. rubra* nodules relative to roots.

Table S2 Expression of symbiosis-related genes in *M. rubra*.

Table S3 *CRRSP* gene expression (FPKM/RPKM) in different plants.

References

1. Rutten, L. *et al.* Duplication of symbiotic lysin motif receptors predates the evolution of nitrogen-fixing nodule symbiosis. *Plant Physiol* **184**, 1004-1023 http://dx.doi.org/10.1104/pp.19.01420 (2020).
